# Outcomes of ‘in‐house’ genetic testing within a specialist hereditary colorectal cancer registry

**DOI:** 10.1111/codi.70640

**Published:** 2026-09-28

**Authors:** Manasawee Srisuttayasathien, Victoria Cuthill, Menna Hawkins, Ashish Sinha, Susan Clark, Andrew Latchford, Kevin J. Monahan

**Affiliations:** ^1^ The St Mark's Centre for Familial Intestinal Cancer St Mark's: The National Bowel Hospital, Central Middlesex Hospital Site London UK; ^2^ Department of Surgery and Cancer Imperial College London London UK

**Keywords:** hereditary colorectal cancer, Lynch syndrome, mismatch repair deficiency, polyposis

## Abstract

**Aims:**

Approximately 5%–10% of colorectal cancer (CRC) cases are due to known Mendelian syndromes. This study aimed to report the diagnostic yield of constitutional genetic testing, alongside clinicopathological factors for hereditary CRC, within a specialised National Bowel Hospital, and outside traditional genetics referral pathways.

**Method:**

This study retrospectively reviewed clinical, pathological and genetic factors using prospectively collected data from the St Mark's Hospital Centre for Familial Intestinal Cancer registry. Between December 2021 and June 2023, consecutive patients at risk of hereditary CRC were selected for genetic testing according to UK National Genomic Testing criteria. The diagnostic yield of genetic testing was calculated by indication. Statistical analysis for clinicopathological data was performed using the Mann–Whitney *U* test, chi‐square test and logistic regression.

**Results:**

A total of 283 consecutive patients underwent genetic testing, 100 (35.3%) mainstreamed with CRC, 95 (33.6%) with multiple polyps, 74 (26.6%) had cascade testing (within families where the probands were known to the registry) and other testing including ‘unaffected’ patients with a relevant family history. Variants were detected in 85 of 283 (30%) patients with known CRC predisposition genes. Diagnostic yields were high for deficient mismatch repair (dMMR) cancer with Lynch syndrome (LS) at 45%, and also for multiple adenoma cohorts at 16%; and in CRC patients under 40 years (irrespective of tumour MMR status) at 16%.

**Conclusion:**

Genetic testing performed by our specialist unit provides patients with a high‐yield, and effective genetic diagnosis, directly indicating comprehensive lifelong care, outside the context of a traditional genetics service.

**What does this paper add to the literature?**

A holistic balancing of unmet needs and clinically actionable results is needed to define genetic testing criteria. This study assesses the diagnostic yield of each criterion based on the national genomic directory for hereditary colorectal cancers, including universal tumour MMR testing for Lynch syndrome. This demonstrates the effectiveness of genetic testing ‘mainstreamed’ outside of traditional clinical genetics models, and elements of this service could be replicated by other colorectal/gastroenterology departments to improve access to patients at risk, and link this testing to other relevant clinical care related to colorectal disease.

## INTRODUCTION

Approximately 30% of colorectal cancer (CRC) cases are due to heritable factors, with 5%–10% due to known Mendelian syndromes, including rare polyposis syndromes and a common disease called Lynch syndrome (LS), which affects 1 in 30 CRC patients [1, 2]. The estimated prevalence of LS is between 1 in 280 and 1 in 400, or about 140,000–200,000 people. Only 10–15,000 diagnoses for LS have been recorded within the National Health Service (NHS), with 95% of persons with LS being unaware of their condition [3]. In the United Kingdom, the management of patients and families at risk adheres to the British Society of Gastroenterology (BSG) guidelines for the treatment, surveillance and prevention of hereditary CRC [4]. Genetic testing falls into three main categories: diagnostic, cascade and criterion‐based for unaffected individuals. Adenoma multiplicity correlates with the diagnostic yield of genetic testing. Most CRCs have adenomatous polyps as their precursor lesions [5]. In a moderate‐sized cohort study, a larger number of adenomas was associated with a greater likelihood of identifying CRC and polyposis predisposition genes. With fewer than 20 polyps, the diagnostic yield of genetic multipanel testing (MGPT) was 7.8%, whereas it was 47.8% for polyps with more than 100 [5, 6]. In the United Kingdom it is recommended that holistic care for polyposis syndromes be offered by centres of expertise within the NHS England Rare Disease Collaborative Network and include colorectal surgeons and gastroenterologists among other specialists [7, 8]. The National Institute for Health and Care Excellence has approved universal testing for all individuals with CRC using immunohistochemistry (IHC) or microsatellite instability (MSI) [9]. This testing identifies tumours with deficient DNA mismatch repair (dMMR) and informs further testing for LS In England, diagnostic genetic testing is shifting from genetic to routine oncology clinics and other specialities, known as ‘mainstream’ [3]. Traditionally, referrals to local genetic services were slow due to unclear assignment to the genetic centre and service bottlenecks. The GMSA National LS transformation project integrates cancer genetic testing into the local cancer team; therefore, there has been a rapid expansion of mainstream services in the past 3–4 years [10, 11]. Since 2021, 276 LS teams have been appointed and trained nationwide across CRC and Endometrial cancer (EC) multidisciplinary team (MDT) meetings. Tumour Mismatch Repair (MMR) testing rates have significantly increased for CRC (43%–94%) and EC (19%–94%), contributing to the rise in new LS diagnoses from 545 cases in 2020 to 1394 cases in 2024 [12].

This review analyses genetic testing results in a highly specialised hereditary colorectal service, which has longstanding expertise in this area. In this study, we planned to analyse the diagnostic yield of constitutional testing according to each clinical criterion of the national genomic directory in LS, inherited polyposis and early‐onset CRC panel, and demonstrate the implementation of real world ‘mainstream’ genetic testing outside traditional clinical genetics models.

## MATERIALS AND METHODS

This was a clinical service evaluation from the St Mark's Hospital Centre for Familial Intestinal Cancer (SMCFIC), London, UK. The study population consists of consecutive patients who underwent genetic testing for hereditary CRC between December 2021 and June 2023. This institution is a national and regional referral centre for hereditary CRC syndromes, supported by an electronic registry. Referred patients complete comprehensive three‐generational family history questionnaires.

The inclusion criteria were consecutive patients who underwent constitutional MMR testing, patients with multiple polyps or those with a family history that fulfilled the NHS GTD criteria [13]. Patients consented to undergo genetic testing; those who did not were excluded from the study. The details of tumour testing and the steps followed to identify patients for constitutional genetic testing are in the previous literature [10].

Genetic data were extracted from the Genomics Laboratory Hub (GLH) online portal for genetic results, and clinicopathological data were obtained from the FilemakerPro© database, which supports the SMCFIC Registry and Epro© electronic patient record (EPR). Patients' family histories were gleaned from their family pedigree, questionnaires and clinical records. Data accuracy was cross‐checked among all databases. Clinicopathological data recorded included age of diagnosis, cancer location, stage at diagnosis, type of surgery, synchronous colonic polyp, extra‐colonic cancer, personal history of smoking and drinking alcohol, histological cell types, tumour differentiation and MMR status. Patients were managed according to the BSG hereditary CRC guidelines, and any deviations from these guidelines were discussed at weekly LS multidisciplinary team meetings (MDT). The genes included in each test panel were set by the national genomic directory, a protocol for NHS England and genomics testing is provided by the North Thames Genomic Laboratory Hub [13].

Through universal screening, all diagnosed CRC patients with tumours that show loss of immunohistochemical markers *MSH2, MSH6 and PMS2*, as well as loss of *MLH1* who have *BRAF* V600E and/or *MLH1* promoter hypermethylation tests suggestive of LS, and who meet clinical criteria, undergo constitutional testing for inherited MMR deficiency, known as UK directory R 210 panel, which includes *MLH1, MSH2, MSH6, PMS2* and *EPCAM*. For polyposis, patients who meet a clinical criterion will undergo constitutional testing for a pan‐colorectal R211 gene panel, including *APC, MUTYH, SMAD4, BMPR1A, STK11, NTHL1, PTEN, MLH1, MSH2, MSH6, PMS2, EPCAM, POLD1, POLE, RNF43 and GREM1*, or the former panel R209, including *APC, MUTYH, SMAD4, BMPR1A, STK11, NTHL1, PTEN, MLH1, MSH2, MSH6, PMS2, EPCAM, POLD1, POLE*. The R210 and R211 panels are nationally agreed tests that link to a crowdsourcing tool, allowing the scientific community to share, download, view and evaluate gene panels [13].

All genetic results were received from NHS GLHs. Target enrichment sequencing, multiplex ligation‐dependent probe amplification (MLPA) and/or Sanger sequencing were tested for the gene panels. Only P/LP variants were interpreted as disease‐causing variants, given VUSs lack sufficient evidence of causation [14]. Sanger sequencing and MLPA were conducted for family variant analysis in predictive testing.

### Statistical analysis

Stata Version 16.1E was used for the statistical analysis. Descriptive statistics were used to describe patients' characteristics, indications for genetic testing and clinicopathological findings. The diagnostic yield was defined as the number of participants who detected pathogenic(P) or likely pathogenic (LP) variants of the CRC‐susceptibility gene, related to the number of participants detected by each screening method. The clinicopathological features of dMMR and pMMR tumours were compared using the Mann–Whitney *U* test and the chi‐square test. The OR was calculated using logistic regression to examine the relationship between clinical and familial risk factors and LS diagnosis. *p*‐Values of <0.05 and a 95% CI were used to define statistical significance.

## RESULTS

### Population characteristics

A summary of baseline characteristics of 283 patients presenting for genetic testing at the LS, Family Cancer and Polyposis Registry, is presented in Table 1. The median age of the patients at the time of genetic testing in our service was 47 years, (range 3–90). Males and females were almost equal, at 53% and 47%, respectively. The average number of probands' first‐degree relatives (FDR) was 6.1. The median age of diagnosis of cancer was 47.5 years. In patients with CRC, the median age of the earliest cancer in the family was 40 years. The FDRs of most CRC patients showed an absence of CRC or other cancers.

**TABLE 1:** Patient characteristics.

Characteristics	Frequency
Total *N*	283
Gender	
Male	150 (53%)
Female	133 (47%)
Median age (range)	47 (3–90)
Median age of cancer at diagnosis (range)^a^	47.5 (32–81)
Median earliest age of cancer in the family^a^	40 (35.5–53)
Median number of patients with CRC having FDR with CRC^a^	0 (0–1)
Median number of patients with CRC having FDR with other cancers^a^	0 (0–3)
Average no of FDR (min–max)	6.1 (2–22)
Patient categorisation	
CRC patients	100 (35.3%)
Patients with multiple adenomas	95 (33.6%)
Cascade testing	74 (26.2%)
Patients with non‐CRC phenotypes	7 (2.5%)
Unaffected testing	3 (1.1%)
Carrier testing for partners of *MUTYH*	4 (1.4%)
Genetic testing categorisation	
Unknown family variant^b^	
R210 panel	21
R211 panel	171
R209 panel	9
R212: STK 11 single gene sequencing	4
Custom testing panels do not match R‐code	4
Predictive testing for known family variants: NGS, Sanger Sequencing, MLPA	83
Constitutional variants' classification^a^ ^,^ ^c^	
Pathogenic (P)	65 (23.0%)
Likely pathogenic (LP)	16 (5.7%)
Variants of uncertain significance (VUS)	7 (2.5%)
No variant detected	198 (70.0%)

Abbreviations: CRC, colorectal cancer; FDR, first‐degree relatives.

^a^ Cohort of 100 CRC patients.

^b^ Few patients were tested on more than 1 panel.

^c^ 3 patients found 1 pathogenic and 1 VUS variant were counted twice.

All cohorts were categorised as mainstream CRC (35.3%), patients with colorectal polyps (33.6%), cascade testing (26.2%), patients presenting with extra‐colonic phenotypes (2.5%), unaffected testing (1.1%) and carrier testing for a *MUTYH* partner (1.4%).

Patients with unknown family variants underwent multigene panel testing (MGPT). The R210 panel was tested in 21 patients, R211 in 171 patients, with other MGPTs in 8 patients. Predictive testing for known family variants was tested for a specific locus in 83 patients. A few patients were tested for multiple panels, guided by the MDT, due to high clinical suspicion and a family history.

Of the patients, 28.7% were positive for genetic testing, as they were found to have pathogenic (P) or likely pathogenic (LP) variants of CRC‐susceptibility genes, and 2.5% had variants of uncertain significance (VUS). In comparison, 70% did not identify a known Mendelian inheritance syndrome.

### Constitutional testing results

The constitutional genetic testing in our study was aligned with the national genomic directory criteria for the LS panel, inherited polyposis, early‐onset CRC panel and predictive testing, and in a few patients was also based on MDT recommendations. Table 2 shows the diagnostic yields based on the criteria.

**TABLE 2:** Diagnostic yields.

The UK National Genomic Testing Directory criteria	Prevalence of hereditary CRC
	(P/LP variants)
All patients with CRC	26/100 (26%)
dMMR tumours	17/38 (45%)
*MLH1*	9/38 (24%)
*MSH2*	4/38 (11%)
*MSH6*	2/38 (5%)
*PMS2*	2/38 (5%)
CRC under age 40	6/38 (16%)
Homozygous *MUTYH*	1/38 (3%)
Compound heterozygous *MUTYH*	1/38 (3%)
*MLH1*	1/38 (3%)
*MSH2*	2/38 (5%)
*PMS2*	1/38 (3%)
All patients with multiple adenomas	15/95 (16%)
5 adenomas under age 40	0/3 (0%)
5 adenomas and CRC	0/15 (0%)
5 adenomas under age 60 and an FDR of 5 polyps or CRC under 60	0/9 (0%)
10 adenomas under age 60	3/35 (9%)
Biallelic/compound heterozygous *MUTYH*	2/35 (6%)
*PTEN*	1/35 (3%)
20 adenomas at age 60 or older	7/24 (29%)
*APC*	1/24 (4%)
*BMPR1A*	1/24 (4%)
*MSH6*	1/24 (4%)
*PTEN*	1/24 (4%)
Biallelic/compound heterozygous *MUTYH*	2/24 (8%)
Compound heterozygous *NTHL1*	1/24 (4%)
5 serrated lesions and ≥2 larger than 10 mm	0/9 (0%)
20 serrated lesions	0/9 (0%)
5 hamartomatous polyps	0/4 (0%)
Other indications based on MDT	1/12 (8%)
*GREM1*	1/12 (8%)

Cascade testing	P/LP/VUS variants
Gene variants detection	37/74
*APC* ^a^	9
*MLH1*	11
*MSH2*	4
*MSH6*	1
*PMS2*	4
Biallelic *MUTYH*	2
*POLE*	2
*SMAD4*	2
*STK11* ^b^	2

Abbreviations: CRC, colorectal cancer; dMMR, deficient DNA mismatched repair; FDR, first‐degree relatives; LP, likely pathogenic; MDT, multidisciplinary team meetings; P, pathogenic; VUS, variant of uncertain significance.

^a^1 patient has *APC* pathogenic variant and secondary finding as *STK11* VUS, it was counted in *APC* ^b^ 1 VUS.

Diagnostic yield overall for CRC patients was 26%, and 16% for the multiple adenomas cohort. For each national genomic testing directory criterion, dMMR tumours had the highest rate of patients with P/LP variants at 45%, followed by 20 adenomas at age 60 or older at 29%, CRC under age 40 at 16% and 10 adenomas under age 60 at 9%. By contrast, no P/LP variants were detected for the other multiple colonic polyp criteria, including five adenomas and a personal history of CRC, five adenomas under age 40, five adenomas under age 60 and the FDR of polyps or CRC, five serrated lesions with two larger than 10 mm, 20 serrated lesions and five hamartomatous polyps. Finally, a few patients who did not meet the national genomic testing directory criteria but were approved for constitutional testing by the MDT were found to test positive at a rate of 8%. The indication for the positive case was mixed polyps, four adenomas and three serrated polyps, in which a *GREM1* pathologic variant was found.

The distribution of gene carriers in dMMR tumours was: *MLH1* at 24% (9/48), *MSH2* at 11% (4/38), *MSH6* at 5% (2/38) and *PMS2* at 5% (2/38). For those CRC under age 40 years, *MSH2* was identified at 5% (2/38), *MLH1* at 3% (1/38), *PMS2* at 3% (1/38), homozygous *MUTYH at 3% (1/38)* and *c*ompound heterozygous *MUTYH* at 3% (1/38). Homozygous plus compound heterozygous MUTYH was the most frequently identified gene in the entire polyposis cohort, at 4% (4/95).

For cascade testing, P/LP/VUS variants were detected 51% for all gene variants tested. LS gene carriers were most prevalent in our clinic, followed by *APC*.

### Clinicopathological characteristics of CRC patients for mismatch repair immunohistochemistry results

In our cohorts, we found that the median age of diagnosis was significantly older in patients with dMMR tumours at 55 years compared to 39 years in pMMR tumours. The location of cancer and the stage of diagnosis significantly differed between the two groups. dMMR tumours were mostly located in the right colon, with fewer cases at stage 4, whereas pMMR tumours were predominantly in the rectum and left colon. Additionally, the presence of histological features of tumour infiltrating lymphocyte (TIL), a Crohn's like lymphocytic reaction, mucinous/signet ring differentiation and medullary growth pattern predicted dMMR tumours.

Table 3 demonstrates the clinicopathological characteristics of CRC patients according to MMR IHC results.

**TABLE 3:** Clinicopathological characteristics of colorectal cancer patients according to mismatch repair status.

Variables	dMMR	pMMR	*p*‐Value
Median age of diagnosis	55 (43–66)	39 (37.5–62)	0.003*
Male/female	22/16	20/28	0.135
Cancer localisation			0.034*
Small bowel	1	0	
Right colon	18	9	
Left colon	10	14	
Rectum	7	20	
Synchronous cancer	2	2	
Any extra‐colonic cancer			0.151
Presence	6	3	
Absence	32	45	
Stage at diagnosis			0.009*
I	7	13	
II	19	8	
III	9	17	
IV	1	6	
Tumour differentiation			0.808
Well	2	3	
Moderate	25	30	
Poor	4	3	
MSI‐H histopathological features: TIL, Crohn's like lymphocytic reaction, mucinous/signet ring differentiation or medullary growth pattern			0.030*
Presence			
Absence	11	5	
	26	41	

Abbreviations: dMMR, deficient DNA mismatched repair; MSI‐H, microsatellite instability‐high; pMMR, proficient DNA mismatched repair; TIL, tumour‐infiltrating lymphocytes.

* Statistically significant.

### Prediction of Lynch syndrome

For CRC patients, 38% of tumours were dMMR, 48% were pMMR and 14% were unknown. The diagnostic yield of LS was 20% for all patients undergoing an MMR IHC test. Table 4 shows the total number of patients undergoing MMR IHC testing and constitutional MMR testing in the CRC cohort.

**TABLE 4:** Total number of patients undergoing mismatch repair immunohistochemistry and constitutional mismatch repair testing in the colorectal cohort.

CRC cohort	*N* = 100
dMMR tumours	38 (38%)
pMMR tumours	48 (48%)
Unknown MMR	14 (14%)
Positive P/LP variants for LS	
dMMR	17/38
pMMR	0/48
Diagnostic yield of LS	20% (17/86)

Abbreviations: CRC, colorectal cancer; dMMR, deficient DNA mismatch repair; LP, likely pathogenic; LS, Lynch syndrome; MMR, DNA mismatch repair; P, pathogenic; pMMR, proficient DNA mismatch repair.

Ten clinicopathological features were included in a logistic regression analysis for the diagnosis of LS. Table 5 shows us that in a tumour on the right side of the colon, the presence of any MSI‐H morphologies, a family history fulfilling Amsterdam criteria or having one or more FDR with CRC or any LS‐related tumours are independent factors for identifying P/LP variants of LS‐susceptibility genes.

**TABLE 5:** Logistic regression of clinicopathological predictors of positive Pathogenic/Likely Pathogenic variants of Lynch syndrome‐susceptibility genes in mainstream cancer patients.

Variables	Odds ratio	95% CI	*p*‐Value
Male	1.47	0.93–7.83	0.066
Age at diagnosis	1.01	0.97–1.04	0.606
Right side of the colon	3.69	1.27–10.71	0.016*
Presence of synchronous colonic polyps	4.86	0.59–40.38	0.143
Presence of any MSI‐H histology	17.29	4.89–61.15	0.000*
Localised cancer (Stage I, II, III)	0.65	0.07–5.74	0.696
Smoking	2.22	0.46–10.80	0.322
Fulfilled Amsterdam II criteria	4.21	1.25–14.15	0.020*
One or more FDRs with CRC or any LS‐related tumours	3.31	1.05–10.43	0.041*

Abbreviations: CRC, colorectal cancer; FDR, first‐degree relatives; LP, likely pathogenic; LS, Lynch syndrome; MSI‐H, microsatellite instability‐high; P, pathogenic.

* Statistically significant.

## DISCUSSION

Hereditary cancer diagnosis and risk assessment with MGPT is moving into the mainstream of clinical practice. Diverse genetic aetiology and clinical phenotype provides a challenge for colorectal and gastroenterology services; however, our data demonstrates this can be delivered effectively outside the context of traditional genetics services.

Mainstream testing for LS has the highest yield to detect CRC‐susceptibility genes at 45%. For inherited polyposis and early‐onset CRC, P/LP variants were detected as the highest in 20 adenomas at age 60 or older at 29%, followed by CRC age <40 at 16% and 10 adenomas under age 60 at 9%. Interestingly, there were no clinically actionable constitutional P/LP variants identified in patients with fewer than 10 adenomas, regardless of age and/or polyp size. Although three P/LP variants were detected from the indication of five adenomas and personal history of CRC, the variants were heterozygous *for the MUTYH gene*, where the risk of a MUTYH carrier does not meet the surveillance eligibility criteria in the United Kingdom [4] or United States [15].

MGPT offers the benefit of investigating genes with phenotypes that remain inadequately characterised, but difficulties lie in management and patient counselling. MGPTs also complicate cascade testing for VUS if the family history is highly specific to the phenotypes, as we had one patient in our cohort who had *an STK11* VUS variant with two FDRs with Peutz‐Jeghers syndrome. These emphasise the importance of the MDT for these inconclusive results and management beyond the scope of the current best practice guidelines. Additionally, molecular tumour boards are an integral component of our MDTs, involving the interpretation of constitutional variants, somatic variants and oncological management. Not only is this a personalised approach to the patient, but it also contributes to national consensus meetings to deliberate on the formulation of guidelines for a novel cancer predisposition gene [16]. Compared to traditional genetic testing in CRC, mainstream services and MDTs enable mainstream physicians to access regional genomic expertise and plan patient management without delay. This is in contrast to inefficient and lengthy external referral process to genetic clinics. International studies have described specific factors that facilitate the success of mainstream genetic services, including extended knowledge and skills training, strong leadership and networked support [17]. One barrier is the lower level of autonomy among non‐geneticists, according to a Genturis survey within public health care in 21 European nations. Predictive tests and formal genetic counsellors are still supervised by clinical geneticists, even though multiple medical specialists in most countries are authorised for mainstream testing. In addition, there is little data about the equity of access or availability of mainstreaming across these health systems [18].

In our service, not only do the patients benefit from genetic testing, but their relatives will also be called for cascade testing before the age of cancer penetrance. Our study found that the average number of FDR patients in our clinic was six, and the predictive testing will be positive for constitutional variants in about half of these for dominantly inherited condition such as LS and FAP. Therefore, detecting one patient with hereditary CRC will prevent three presymptomatic relatives from developing CRC.

Data from our cohort have shown the apparent benefit of universal MMR testing, with 20% diagnostic yield in detecting LS carriers among our cancer patients. The diagnostic yield in our study was far higher than in previous studies because the CRC patients in our population are highly selective patients suspected of hereditary cancer by referral [19, 20, 21].

The age of patients with dMMR tumours was not lower than that of patients with pMMR tumours. This was consistent with an earlier study [22]. The dMMR tumours in our study exhibit distinct clinicopathological features, including a higher incidence of localisation to the right side of the colon, MSI‐H morphologies and a reduced tendency to metastasise. As the right colon is widely considered the most immunoreactive section, these features are known to have a better prognosis and suggest a good response to immunotherapy [23, 24].

Clinical data, tumour characteristics and family history factors were evaluated using a logistic regression model to predict LS. We did not observe a higher prevalence of LS in males with CRC than in their female counterparts, in contrast to the PREMM5 model, which employs personal and family cancer histories to forecast the likelihood of carrying a gene in LS [25]. Also, the age of diagnosis of CRCs did not affect the probability of LS when approached from the bottom up. Despite the fact that LS carriers have a higher risk of CRC at an early age, this does not imply that CRC patients at older ages are sporadic. Additionally, it was found that tumours at the right‐sided colon, the presence of any MSI‐H morphologies, the family history that fulfilled Amsterdam criteria or having one or more FDR with CRC or any LS‐related tumours were significantly related to P/LP variants of LS‐susceptibility genes. In the United Kingdom, it is possible to combine these tumour characteristics with personal and familial cancer histories to create future LS predictive tools, as LS is primarily a tumour‐first approach.

As hereditary CRC is rare, a retrospective clinical review may clarify the causal relationship between genetic variants and clinical phenotype [26]. However, the limitations of it stem from the nature of retrospective data collection, including some clinical and pathological data that were incomplete, as well as a bias towards known family variants in a few patients who also met the clinical criterion for genetic testing. This study may not represent the diagnostic yield of genetic testing in all CRC patients because our study site is highly specialised and may result in ascertainment bias in the population tested. Given the limited time period for the evaluation, the number of rare indications is small, preventing meaningful conclusions. In addition, gene panel lists in the UK have also been added over time. Some patients with clinically indicated conditions will be re‐analysed, but most were not offered this service; therefore, our study is based on the gene panels list at the time of testing.

## CONCLUSIONS

This study reported the diagnostic yield of each indication for constitutional testing, based on the UK National Genomic Directory, among patients at risk of hereditary CRC. Genetic testing is feasible in a specialised gastrointestinal clinic as a mainstream colorectal disease and cancer service. LS is the most prevalent among inherited CRCs. Cascade testing effectively identifies individuals who are carriers of hereditary CRC genes, facilitating prevention and early detection.

## AUTHOR CONTRIBUTIONS

**Manasawee Srisuttayasathien:** Data curation; writing – review and editing; formal analysis; investigation; methodology; project administration; writing – original draft; visualization. **Victoria Cuthill:** Data curation; resources. **Menna Hawkins:** Data curation; resources. **Ashish Sinha:** Writing – review and editing. **Susan Clark:** Writing – review and editing. **Andrew Latchford:** Writing – review and editing; validation. **Kevin J. Monahan:** Conceptualization; writing – review and editing; supervision; methodology; formal analysis; funding acquisition.

## FUNDING INFORMATION

No funding was received for this article.

## CONFLICT OF INTEREST STATEMENT

The authors report there were no conflicts of interest and that no financial support was received.

## ETHICS STATEMENT

This is part of a service evaluation for which approval was obtained from London North West Hospitals NHS Trust.

## Data Availability

The data that support the findings of this study are available on request from the corresponding author. The data are not publicly available due to privacy or ethical restrictions.
